# Supplementary material for: Deducing genotypes for loci of interest from SNP array data via haplotype sharing, demonstrated for apple and cherry
Source: PLoS One. 2023 Feb 7;18(2):e0272888. doi: 10.1371/journal.pone.0272888 (PMC9904487; doi:10.1371/journal.pone.0272888)
Supplement: S3 Table — Sharing via IBD is shown in bold, otherwise sharing was via IBS. Individuals annotated with an asterisk (*) are ancestral sources of alleles. For H13, ‘Windsor’ and ‘Venus’ shared the same extended haplotypes with ‘Blackheart’ and ‘PMR-1’, so they are listed under H13 for both ancestral sources. (DOCX) [file pone.0272888.s003.docx]

**S3 Table: Alleles deduced for the *R_f_* locus for sweet cherry cultivars and selections by haplotype sharing.** Sharing via IBD is shown in **bold**, otherwise sharing was via IBS. Individuals annotated with an asterisk (*) are ancestral sources of alleles. For H13, ‘Windsor’ and ‘Venus’ shared the same extended haplotypes with ‘Blackheart’ and ‘PMR-1’, so they are listed under H13 for both ancestral sources.

| **Haplotype name from Sandefur et al. (2016) (ancestral source)** | **Cultivars with shared haplotypes at the locus (length of extended shared haplotypes in cM)** |
| --- | --- |
| r_f_ (Blush) | |
| H1 (Ambrunes) | **Ambrunes*** (60.2) |
| H1 (Bertiolle) | **Bertiolle*** (60.2) |
| H1 (Emperor Francis) | **Emperor Francis*** (60.2) |
| H1 (Empress Eugenie) | **Empress Eugenie***, **Van**, **Newstar** (60.2), **Summit** (46.1), **BB** (15.32), **Lapins** (36.1), **GG** (20.2), **Sweetheart** (32.8), **Rainier** (32.2), Windsor, Venus (19.4) |
| H1 (Napoleon) | **Napoleon***, **Gil-Peck**, **Satonishiki**, (60.2), **Olympus**, **Lambert** (25.2), **Stella**, **Benton**, **Cowiche** (16.7) |
| H1 (Schmidt) | **Schmidt*** (60.2) |
| H2 (Napoleon) | **Napoleon***, **Black Republican**, **Bing**, **Chinook**, **Index**, **Selah**, **Vic** (40.0), **AA**, **Brooks**, **CC**, **DD**, **EE**, **JJ, Rainier** (15.1) |
| H3 (Schneiders) | **Schneiders***, **Regina** (60.2) |
| H4 (Cristobalina) | **Cristobalina*** (60.2) |
| H5 (Emperor Francis) | **Emperor Francis*** (60.2) |
| H6 (Dzherlo) | **Dzherlo*** (60.2), Krupnoplodnaya (14.2) |
| H7 (MIM 17) | **MIM 17*** (60.2) |
| H7 (MIM 23) | **MIM 23*** (60.2) |
| R_f_ (Mahogany) | |
| H9 (Black Republican) | **Black Republican***, **Bing** (60.2), **Van** (41.4), **Olympus** (31.5), **Lapins** (29.9), **Sweetheart** (27.5), Regina (20.7) |
| H10 (Schmidt) | **Schmidt***, **Vic** (60.2) |
| H10 (Summit) | **Summit***, **Sunburst** (60.2) |
| H11 (Early Burlat) | **Early Burlat*** (60.2), Moreau, Chelan (55.3), **Cashmere** (53.8), **Kiona** (50.1), Benton (36.6), **Cowiche** (35.3), **Tieton** (32.8), **Brooks** (29.6) |
| H13 (Blackheart) | **Blackheart***, **Lambert**, **Stella**, **Cashmere**, **Index**, **Newstar**, **Tieton** (60.2), **Glacier**, **Selah** (33.3), **Chelan** (29.8), Venus (23.4), Windsor (18.3) |
| H13 (PMR-1) | **PMR-1***, **AA** (60.2), **EE** (53.8), **BB** (41.4), **JJ** (35.2), Venus (23.4), Windsor (18.3) |
| H14 (Ambrunes) | **Ambrunes*** (60.2) |
| H14 (Cristobalina) | **Cristobalina*** (60.2) |
| H14 (Bertiolle) | **Bertiolle*** (60.2) |
| Pedigree or Flanking Haplotypes Necessary | |
| r_f_ (Blush) | |
| H8/17: (MIM 17) | **MIM 17*** (60.2) |
| H8/17: (MIM 23) | **MIM 23*** (60.2) |
| R_f_ (Mahogany) | |
| H8/17 (PMR-1) | **PMR-1***, **CC** (60.2), **GG** (50.4), **DD** (38.7), Moreau (39.8) |
| H8/17 (Dzherlo) | **Dzherlo*** (60.2), Krupnoplodnaya (32.1) |
| H8/17 (Early Burlat) | **Early Burlat*** (60.2), **Glacier**, **Kiona** (53.8) |

For paper:

**Deducing genotypes for loci of interest from SNP array data via haplotype sharing, demonstrated for apple and cherry**

by Alexander Schaller, Stijn Vanderzande, Cameron Peace
