# Supplementary material for: Deducing genotypes for loci of interest from SNP array data via haplotype sharing, demonstrated for apple and cherry
Source: PLoS One. 2023 Feb 7;18(2):e0272888. doi: 10.1371/journal.pone.0272888 (PMC9904487; doi:10.1371/journal.pone.0272888)
Supplement: S4 Table — Sharing via IBD is shown in bold, otherwise sharing was via IBS. Individuals annotated with an asterisk (*) are ancestral sources of alleles. (DOCX) [file pone.0272888.s004.docx]

**S4 Table: Alleles deduced for the GD12 locus for various apple cultivars and selections by haplotype sharing.** Sharing via IBD is shown in **bold**, otherwise sharing was via IBS. Individuals annotated with an asterisk (*) are ancestral sources of alleles.

| **Allele (ancestral source)** | **Cultivars with shared haplotypes at the locus (length of extended shared haplotypes in cM)** |
| --- | --- |
| 153 (Winesap) | **Winesap***, **Delicious**, **Gala**, **Kidd’s Orange Red**, **Melrose**, **Scired**, **Splendour**, **WSU 2**, **WSU 5** (71.9), **Blushing Golden** (70.4), **BC 8S-27-43** (60.5), **Nicola** (55.1), **Aurora Golden Gala** (45.2), **Co-op 17**, **Goldrush** (44.2), **Sciros** (44.2), **BC 8S-27-43** (41.1), **NY 752**, **NY 88** (37.5) |
| 155 (Ben Davis) | **Ben Davis*** (71.9), **Cortland** (62.9) |
| 155 (Beauty of Bath) | **Beauty of Bath***, **Discovery** (71.9) |
| 155 (Cox’s Orange Pippin) | **Cox’s Orange Pippin***, **James Grieve**, **Kidd’s Orange Red** (71.9), **Ingrid Marie** (49.0), Fortune (47.3), **Lord Lambourne** (45.0), Jonathan, Linda (25), **Elstar** (21.3) |
| 155 (Esopus Spitzenburg) | **Esopus Spitzenburg***, **Jonathan** (71.9), **Jonafree** (63.5), **Haralson**, **Honeygold**, **Liberty**, **State Fair, Wealthy** (52.5), **Regent** (49.0), **Minnewashta** (46.2), **Melrose** (45.0), **Kerr**, **Oriole** (37.8), **Arlet**, **Idared**, **Fiesta** (35.1), **Akane**, **Autumn Crisp**, **Delorgue**, **Empress**, **Jonamac**, **Monroe**, NY 65707-19, **NY 88**, **Sansa**, **Sawa**, **Tsugaru** (24.9), State Fair (18.1) |
| 155 (Granny Smith) | **Granny Smith***, **Lady Williams**, **Cripps Pink**, **Cripps Red** (71.9) |
| 155 (Malinda) | **Malinda** (71.9) |
| 155 (McIntosh) | **McIntosh***, **Empire**, **PRI 1661-2**, **Spartan** (71.9), **Macoun**, **Jonamac** (37.6), **Burgundy** (20.7) |
| 155 (Northern Spy) | **Northern Spy***, **Keepsake** (55.1), Fireside (37.1), **Honeycrisp** (21.4), Co-op 18 (9.8) |
| 155 (Northern Spy) | **Northern Spy**, **Sweet 16** (71.9) |
| 155 (Wagener) | **Wagener** (71.9) |
| 155 (Worcester Pearmain) | **Worcester Pearmain***, **Discovery** (71.9), Topaz (38.9), **Akane** (37.6), Clivia, Pinova (34.2), James Grieve (22.3), GMAL 4327, GMAL 4328, GMAL 4329 (12.1) |
| 155 (Worcester Pearmain) | **Worcester Pearmain** (71.9), **Lord Lambourne** (50.4), |
| 155 (UP_Golden Delicious) | **Golden Delicious***, **Autumn Crisp**, **Ginger Gold**, **Tsugaru** (71.9), **Honeycrisp** (70.3), Cameo (66.5), **Sunrise**, **Silken** (53.8), **Cripps Red** (42.6), **Ambrosia** (38.2), **Elstar** (37.6), **Goldrush** (34.3) |
| 155 (Lady Williams) | **Lady Williams*** (71.9) |
| 155 (Wagener) | **Wagener*,** **Idared** (71.9) |
| 155 (Yellow Transparent) | **Yellow Transparent***, **Lodi**, **Early Cortland**, **Ginger Gold** (71.9) |
| 157 (Esopus Spitzenburg) | **Esopus Spitzenburg** (71.9) |
| 157 (Montgomery) | **Montgomery*** (71.9) |
| 157 (Rome Beauty) | **Rome Beauty*** (71.9), **Monroe** (38.2), **Burgundy** (23.5), Liberty, Macoun (9.7) |
| 157 (Russian Seedling) | **Russian Seedling*** (71.9) |
| 157 (Yellow Transparent) | **Yellow Transparent*** (71.9) |
| 157 (Beauty of Bath) | **Beauty of Bath*** (71.9) |
| 159 (Ben Davis) | **Ben Davis*** (71.9), F_2_26829-2-2 (16.5), PRI 14-126 (16.5) |
| 159 (Granny Smith) | **Granny Smith** (71.9) |
| 159 (UP_Delicious) | **Delicious***, **Cameo**, **Empire**, **PRI 1661-2**, **Enterprise**, **Spartan**, **NJ 90** (71.9), **NY** **65707-19** (61.5), **Co-op 15** (47.4), **NJ 90** (46.8), **Fuji** (37.2), **Braeburn**, **Sonya** (36.7), **Ambrosia** (34.3) |
| 159 (Cox’s Orange Pippin) | **Cox’s Orange Pippin*** (71.9) |
| 159 (Malinda) | **Malinda***, **Haralson** (71.9) |
| 159 (Winesap) | **Winesap*** (71.9) |
| 161 (Russian Seedling) | **Russian Seedling*** (71.9) |
| 161 (Duchess of Oldenburg) | **Duchess of Oldenburg*** (71.9), **Wealthy**, **Fireside**, **Linda**, **Fantazja,** **Sawa** (38.2) |
| 187 (UP_Braeburn) | **Braeburn*** (71.9), **Scifresh** (52.5) |
| 187 (McIntosh) | **McIntosh***, **Fantazja** (71.9), **Melba** (61.8), Dayton (47.3), **Cortland**, **Early Cortland**, **Regent** (44.2), Vista Bella (29.7), Prima (25.9) |
| 187 (Montgomery) | **Montgomery***(71.9), **Lodi** (34.9) |
| 195 (Grimes Golden) | **Golden Delicious***, **Arlet**, **Cripps Pink**, **NY 752**, **Honeygold**, **Silken**, **Splendour**, **Chinook**, **Scired**, **Sciros** (71.9), **Pinova** (61.9), **Blushing Golden** (57.3), Dayton (55.7), **Delblush** (51.1), **Delblush** (48.5), **Topaz** (48.5), **PRI 14-126** (45.2), **Gala**, **Chinook**, **Nicola**, **NY 543**, **Sansa**, **Sonya**, **WSU 2** (42.5), **Aurora Golden Gala** (42.4), **Scifresh** (41.9), **Prima** (34.3) |

For paper:

**Deducing genotypes for loci of interest from SNP array data via haplotype sharing, demonstrated for apple and cherry**

by Alexander Schaller, Stijn Vanderzande, Cameron Peace
