# Supplementary material for: Deducing genotypes for loci of interest from SNP array data via haplotype sharing, demonstrated for apple and cherry
Source: PLoS One. 2023 Feb 7;18(2):e0272888. doi: 10.1371/journal.pone.0272888 (PMC9904487; doi:10.1371/journal.pone.0272888)
Supplement: Attachment — Submitted filename: Response to Reviewers .docx [file pone.0272888.s007.docx]

**Response to Reviewers**

**Our responses are provided in bold to each reviewer comment. Line numbers referred to are associated with the clean resubmitted manuscript “Manuscript”).**

Review Comments to the Author

Reviewer 1

Comment 1: “I suggest that the authors briefly comment in the introduction section the case of study used in this manuscript for each type of loci (Ma and Ma3 for acidity in apple; 2. microsatellite marker GD12; and 3. Mendelian trait locus Rf for sweet cherry fruit color). Although the authors included some information in the materials and methods section, it would be helpful to introduce the QTL, MTL and the multi-allelic microsatellite marker used in this manuscript.”

**Response: A paragraph was added to the Introduction at lines 96 to 106 to introduce these loci, moving the information from what was the second paragraph of the Materials and Methods. We hadn’t included this background in the Introduction of original manuscript because we are trying to make the point that the approach is relevant for any locus. Therefore, the study is not about these four specific loci, and we considered that introducing them prior to the Materials and Methods would give the incorrect emphasis to the study. To address the reviewer comment and fulfill our objective, our new Introduction paragraph, we added “for example” and “an example” as the lead-in to mentioning each of the four loci.**

Comment 2: Lines 216-219: I suggest that the authors include the physical position (bp) in the text of the manuscript

**Response: Physical positions of each locus have been added, both (as appropriate) the location of the marker associated with the locus and the bp length that the locus is embedded in or flanked by. Line 244 to Line 251**

3. Line 223: The authors cited in the manuscript the paper that reported the nine important breeding parents. Ideally, they should also include the name of the breeding parents between parenthesis. “Successful deduction of alleles via both IBD and IBS of extended shared haplotypes with ancestral sources of the nine important breeding parents…”

**Response: The nine important breeding parents have been added in parentheses at lines 261-262.**

Reviewer 2:

Comment 1: lines 77-83: this can be re-written a bit more clearly. I see you want to differentiate between high-density SNP arrays (which are mainly used for association mapping analysis and genomic predictions) and the need of trait-targeted and high-throughput genotyping systems for breeding and germplasm management.

**Response: We wanted to make it clear that SNP arrays are only part of the tool set and are utilized mostly for selected, elite individuals. Other tools (e.g., locus-specific DNA tests) are utilized on seedlings, however there isn’t an available approach to effectively connect these two tools together. Therefore, a translational method is necessary. This clarification was added in lines 89-91.**

**“Thus, methods to translate between the outcomes of SNP arrays and locus-specific tests are needed to integrate new and historic data and to integrate data across various germplasm levels.”**

Comment 2: lines 83-91: again, I think this can be simplified a little. While newly developed SNP arrays can include validated markers linked to traits of interest, your objective here is to develop a method to identify samples carrying favorable alleles using SNP array-datasets already generated.

**Response: Our goal is to link existing and historic data sets to SNP array data to allow information to be compared. This section was edited to clarify our goals from lines 89-94.**

Comment 3: lines 140-157: this part is rather confusing. Could you maybe re-write in a clearer way, and provide tables/figures showing the data used and the process applied?

**Response: A new figure (Fig. 1) was included, which describes visually the workflow for identifying the location of a locus, assigning alleles, and then confirming each haplotype pattern is unique. This figure then connects with Fig. 2 (previously Fig. 1). The text was also updated at lines 151-172 to clarify the data used and process applied.**

Comment 4: lines 163-165: does this mean that you used pedigree information to deduce the haplotype patterns? What does it meant "selections with unknown genotypes"? Didn't you use only accessions that had been genotyped with SNP arrays (as from paragraph "Data set")?

**Response: The text was adjusted at lines 191-193 to clarify the meaning, as follows:**

**“Next, the haplotype patterns present in all cultivars and selections in each Crop Reference Set with unknown genotypes for each target locus were compared to the SNP haplotype patterns of the ancestral sources to identify all cases of haplotype sharing (Figure 2).”**

Comment 5: line 198: what about validating with phenotypic data? If the objective is to identify samples carrying favorable alleles to certain traits, it seems important to verify if your method is correctly assigning the deduced alleles.

**Response: Our goal for this manuscript was to deduce alleles for any locus based on SNP data, not to confirm how well the deduced alleles are associated with phenotypes. As mentioned above in response to reviewer 1’s comment 1, our study is focused on the approach and not focused these four particular loci, which were just chosen to be examples for demonstrating the approach. To help clarify the purpose of the study, a sentence was added in the discussion, at lines 417-420, as follows:**

**“While the method was demonstrated in apple and sweet cherry using the locus types represented by *Ma, Ma3, R_f_,* and *GD12*, it could be expanded to other loci, other types of loci, and other crops that have SNP arrays or other genome-wide data available, especially where linkage disequilibrium is high.”**

Comment 6: line 215: maybe a figure here, with chromosome numbers, would be useful.

**Response: This suggested additional useful information has been added as a new figure (Fig. 3).**

Comment 7: lines 293-345: it is really hard to follow all the details reported in the results. I strongly suggest to make more use of figures to show your findings.

**Response: (Original lines 293-345 referred to the section “Identity-by-state among ancestral sources”.) The original text had a paragraph each for the QTs, MTL, and SSR locus, and referred to details provided in S6 Tables. The reviewer’s suggestion was acted upon with the inclusion of a new figure (Fig. 4) that exemplifies how results were obtained, and therefore how the method works, for one of the loci. This new diagram represents visually the first of the cases previously (and again) provided in S6 Tables.**

Comment: line 67: change "tiny" with "small"

**Response: Done**

- line 70: change "numerous" with "several"

**Response: We changed the word to “many”. The original sentence (“In the RosBREED project [22, 32, 33], SNP arrays were developed and used in apple, cherry, and peach on large breeding germplasm sets that were pedigree-connected and included numerous important breeding parents…”) refers to three crops, covering more than several important breeding parents (57 for apple, 38 for peach, and 25 for cherry).**

- line 79: change "transient breeding selections" with "seedlings at early stages of selection"

**Response: Done**

- line 80: I'd remove the sentence "tens of dollars (rather than cents to a few dollars)", since costs are in constant development

**Response: The statement referring to example costs was removed during the editing of this paragraph which is now lines 79-94.**

- lines 84-86: I find the sentence "Instead, genotypes for QTLs, Mendelian trait loci (MTLs), or any loci of interest such as multi-locus SSRs are not the immediate output of SNP arrays and are hidden within a sea of data points." a bit mis-leading, since SNP arrays are used often to identify loci of interest

**Response: The sentence was removed and the entire paragraph, lines 79-94, was edited to clarify.**

- line 140: is this what is shown in Table S2?

**Response: The comment refers to the sentence that was on line 140 of “Reported genotypes of cultivars and their ancestors were assembled for the QTLs, MTL, and SSR.” The answer is yes, and to help clarify for any reader a new table (Table 1) has been added that lists the previously reported genotypic data that was the starting point for each locus for our study.**

- line 141: is this for a total of 121 accessions, as reported in previous paragraph?

**Response: The comment refers to the sentence that was on lines 140-142 of “For *Ma* and *Ma3*, both functional alleles of nine important breeding parents and their ancestral origins were obtained from their previous allocations in [40] and [43].” The answer is yes, and the new table mentioned above has been included to help make this clear for any reader.**

- line 143: 60 cherry accessions?

**Response: The comment refers to the sentence that was on lines 142-143 of “For *R_f_*, both functional alleles assigned to 17 cultivars and traced to their ancestral sources were obtained from [4].” The answer is yes, and again the new table added should help make this clear for any reader.**
